# 

**Published:** 1881-06-20

**Authors:** 


### TABLE OF CONTENTS.

ORIGINAL AND SELECTED ARTICLES.

Remarks upon the Treatment of Pneumonia, by R. C. Word, M.D., Professor of Physiology in the Southern Medical College, 201. A Case of Pleuritic Effusion, by Thomas F. Houston, M.D., Atlanta, Ga., 203. Facts from the Small-Pox Hospital at Troy, New York, by Clarkson C. Schuyler, M.D., Assistant Surgeon, 205. The Constant or Galvanic Current of Electricity in Diseases of the Nervous System, by Edward C. Mann, M.D., New York City, 209. Affections of the Eye caused by Masturbation, by M. Landesberg, M.D., 211. Case of Chronic Dysentery Cured by Large Doses of Ipecacuana, by W. S. Whitwell, M.D., 213. Destruction of the Membrana Tympani and Application of the Toynbee Disc, by A. S. Core, M.D., Quincy, Ill., 214. Neuralgia of the Testis, by Geo Halsted Boyland, A.M., M.D., late Surgeon French Army, etc., 216. Concentrated Solution of Quinia, 217.

ABSTRACTS AND GLEANINGS.

Excision of a Portion of the Stomach and Duodenum, 218. Aphasia; Erysipelas—Case in Practice, 219. Viburnum Prunifolium, 220. Fractures of the Forearm, 221. Hypodermic Injection of Atropiæ Sulphur—A Specific for Sciatica, 222. How to Preserve the Teeth; Sand Bag for the Sick-Room, 223. Morphia and Chloroform Combined, for Anesthesia; Aromatic Tetrachloride of Carbon; Hypodermic Injections of Coffee, 224. Usual Situation of Diphtheritic Membrane; Boracic Acid for Cholera; Heroic Police; Cure for Night Sweats, 225. The Wet-Sheet in Pneumonic Fever, 226. Sodium Phosphate in Habitual Colic, 227. Suit for Damages; Poisonous Ice; Operative Interference in Gunshot Wounds of Peritoneum, 228. Psylium Seed in Constipation; Dangers of Chlorate of Potash; French Treatment of Asthma, 229. Mortality on the Globe; A Triumph of Dentistry; Cascara Sagrada, or Rhamnus Purshiana; Whooping-Cough; Chloral Hydrate for Toothache, 230.

SCIENTIFIC ITEMS.

Trees and Lightning; Refrigeration and Animal Heat, 231. Precocity a Sign of Inferiority; Telegraphing without Wires, 232.

PRACTICAL NOTES AND FORMULÆ.

Treatment of Chronic Cystitis; Ointment for Eczema; Irritable Bladder; Quillaia for Toothache, 233; Soda Mint; For Albuminuria in Pregnancy; Arthritis; Eczema; Syphilitic Neuralgia, 234. Caustic Powder; Cod-Liver Oil; Tully's Powder; For the Stomach; Tartaric Acid in Diphtheria, 235. Ointment of Wormwood; Asthma; Improved Dover's Powder; Preparation for Corns, 236.

EDITORIAL AND MISCELLANEOUS.

Editorial Notices, 237. International Medical Congress; Pamphlets Received; Book Notices, 238. Special Notices, 240.

